# The prevalence and risk factors of work-related musculoskeletal disorders among adults in Ethiopia: a study protocol for extending a systematic review with meta-analysis of observational studies

**DOI:** 10.1186/s13643-020-01403-9

**Published:** 2020-06-08

**Authors:** Tsiwaye Gebreyesus, Kalkidan Nigussie, Moges Gashaw, Balamurugan Janakiraman

**Affiliations:** 1 Department of Physiotherapy, School of Medicine, College of Health Sciences and Ayder Comprehensive Specialized Hospital, Mekelle University, Mek’ele, Ethiopia; 2 Department of Physiotherapy, School of Medicine, College of Medicine and Health Sciences, and Specialized Hospital, University of Gondar, P.O. Box No. 196, Gondar, Ethiopia

**Keywords:** Prevalence, Work-related musculoskeletal pain, Population-based observational studies, Systematic review protocol, Ethiopia

## Abstract

**Background:**

Work-related musculoskeletal disorders impose a significant and most often underappreciated burden to the individual, nation, healthcare system, and society as a whole. To the best of our knowledge, there is a lack of reliable estimates on the prevalence of work-related musculoskeletal disorders in Ethiopia. The objective of this study will be to assess the existing literature on the prevalence rates and determinant factors of work-related musculoskeletal disorders in Ethiopia.

**Method:**

We will search PubMed/MEDLINE, Embase, SCOPUS, PsycINFO, PEDro, and Ebsco (from January 2000 onwards). Gray literature will be identified through searching Google Scholar and dissertation databases. Observational studies reporting on the prevalence of work-related musculoskeletal disorders among adult Ethiopians will be included. The primary outcome will be the prevalence of work-related musculoskeletal disorders. Secondary outcomes will be the prevalence of any risk factors in association with work-related musculoskeletal disorders. Two reviewers will independently screen all citations, full-text articles, and abstract data. The study methodological quality (or bias) will be appraised using an appropriate tool. If feasible, we will conduct random effects meta-analysis of observational data. Subgroup analyses will be conducted to explore the potential sources of heterogeneity (e.g., gender, sample size, type of occupation). Publication bias and heterogeneity between the included studies will also be assessed and reported.

**Discussion:**

This systematic review will provide a synthesis of the literature on the prevalence of work-related musculoskeletal disorders and their risk factors in Ethiopia. The results of this review could help the policymakers in occupational health and healthcare sectors in identifying priority areas for interventions in work-related musculoskeletal disorders and will also serve as a baseline for the decision-making processes of musculoskeletal health promotion, work exposure implementations, and prevention programs in workplaces.

**Systematic review registration:**

PROSPERO, CRD42020164240

## Background

Musculoskeletal disorders (MSDs) are common workplace health problems characterized by a range of symptoms like pain, ache, and discomfort in bodily regions [1, 2]. Musculoskeletal disorders (MSDs) are impairments of body structures such as muscles, tendons, fascia, ligaments, joints, nerves, bones, or a localized blood circulation system either caused or aggravated by poor fitness, and poor health habits, but a major proportion of MSDs are caused by physical work exposures [3, 4]. And these impairment occurs secondary to sudden injuries and cumulative trauma, with the latter being the most common mechanism behind work-related musculoskeletal disorders (WRMSDs) [1, 3, 5].

According to Global Burden of Diseases (GBD), injuries, and risk factors study 2017, between 2007 and 2017, among the three causes which are observed to have resulted in a further increase in the number of all-age years lived with disability (YLDs), low back pain (LBP) is attributable to a further increase of 17,·5% (95% UI 16·2–19·0), While the YLDs counts are heavily concentrated in working-age (i.e., from 20-54 years), a pattern which is particularly evident among causes such as musculoskeletal disorders, mental disorders, and neurological disorders which sum to more than 45% of all YLDs in these age groups [6, 7]. This finding is notable as the working-age group in countries with low socio-demographic index (SDI) has a considerable number of years to live, and musculoskeletal disorders of even lower to moderate disability weights can cause substantial non-fatal burden and lost human capital [8]. Moreover, musculoskeletal disorders being three of the top 10 conditions in terms of disability and burden from non-communicable diseases (NCDs) in developing countries. Total disability-adjusted life years (DALYs) from low back and neck pain also increased, rising by more than 17% from 2000 to 2017 [9–11].

GBD 2017 reported that MSD’s are the second most common cause of years lost to disability, while years of life lost are declining in sub-Saharan. Further, the burden of MSD will only continue to increase as the sub-Saharan population ages [7, 12]. Work-related musculoskeletal disorders (WRMSDs) and other musculoskeletal conditions remain less prioritized and empirically unrepresented in low-middle income countries (LMICs), particularly in Ethiopia due to focus on more pressing and life-threatening health issues like NCDs and infectious diseases [13]. A previous systematic review has however reported on the prevalence of WRMSDs in Africa which included a majority of studies from South Africa and Nigeria. The prevalence of WRMSDs varies from 13 to 92% in South Africa and Ghana respectively [14]. Empirically, racial, economic, and social homogeneity is not a feature of Africa and may underlie the reported difference in the prevalence. Hence, it is logical, therefore, to argue that genetic diversity, differences in social structure, economics, and other Ethiopian specific factors may influence the WRMSDs prevalence among Ethiopians. Further, musculoskeletal disorders remain a major global health concern and an immense burden for LMICs like Ethiopia where health budgets are already constrained and channeled towards the life-threatening conditions. Studies have shown that musculoskeletal pain prevalence among the working population in Ethiopia varies from 35% to 74.5% [13–22].

Though in the past decade many individual studies have reported the prevalence of regional pain, general WRMSDs, and factors associated with the working Ethiopian population, to our knowledge, no published systematic review reporting on the prevalence of work-related musculoskeletal on Ethiopian adult population exists. The consensus of data from this review will help occupational health-related policymakers, healthcare professionals and program managers in developing countries in particular Ethiopia, to gain a better understanding of the prevalence, causes, and trends to build a better evidence-based occupational musculoskeletal health and disorders prevention programs. The objective of this study will be to assess the prevalence of work-related musculoskeletal disorders and their risk factors among the working adult population in Ethiopia.

Specific review questions are the following:

- What is the prevalence of musculoskeletal disorders in the categories of bodily regions, for different occupational settings and subgroups in Ethiopia according to internationally published studies?
- What are the occupational factors and patterns (pain sites, frequency, distribution across body) of musculoskeletal pain, what are the factors that cause and/or related to good or poor musculoskeletal health for different occupational settings and subgroups in the adult working population in Ethiopia according to internationally published studies?
- What is the methodological quality of the prevalence studies, intending to identify approaches to improve future research quality?

## Methods

### Systematic review registration and reporting

The present review protocol has been registered within the PROSPERO database (registration number CRD42020164240) and is being reported in accordance with the reporting guidance provided in the Preferred Reporting Items for Systematic Reviews and Meta-Analyses Protocols (PRISMA-P) statement [23, 24] (see checklist in Additional file 1) and Meta-analysis Of Observational Studies in Epidemiology (MOOSE) reporting guideline [25].

### Data sources and search strategies

The primary source of literature will be a structured search of electronic databases (from January 2000 onwards, as we are primarily interested in the contemporary literature): PubMed/MEDLINE, Embase, SCOPUS, PsycINFO, PEDro, and Ebsco. The secondary source of potentially relevant material will be a search of the gray or difficult to locate literature, including Google Scholar, dissertation databases and other relevant databases (e.g., World Health Organization, Centers for Disease Control and Prevention, Work Place Health Promotion). We will perform hand searching of the reference lists of included studies, relevant reviews or other relevant documents. The search will include a broad range of terms and keywords related to musculoskeletal disorders, workplace and occupation, prevalence and the geographical area ‘Ethiopia’. A draft search strategy for PubMed/MEDLINE is provided in Additional file 2. Only studies published in English (or with an English version of the document) will be eligible. Moreover, the corresponding authors will be contacted by mail whenever a need arise or for any difficulties faced during data extraction.

### Eligibility criteria

Studies will be selected based on the following criteria: participants, condition or outcome(s) of interest, study design, and context.

Participants (population): We will include studies involving adult population (≥ 18 years of age, regardless of sex) working in governmental sectors or private sectors or self-employed in Ethiopia.

#### Condition or outcome(s) of interest

The primary outcome will be the prevalence (e.g., point prevalence, period prevalence, prevalence rate) of WRMSDs (including neck disorders, low back disorders, or other chronic disorders) indicating the number of people that have the disorder divided by the population number at a given point in time, presented often as a (prevalence) proportion. We will use the authors’ reported definitions using standardized scales or questionnaires (e.g., standardized nordic questionnaire, Dutch musculoskeletal questionnaire, or visual analog scale) providing sufficient information to calculate the prevalence. Secondary outcomes will be to identify the risk factors of musculoskeletal disorders (e.g., psychosocial factors, posture, heavy physical work, workplace-related factors, and smoking). When outcome results (e.g., proportion and 95% CI) are not directly reported and it is feasible, we will calculate estimates from the number of cases and sample size mentioned in each single study.

#### Study design and context

Eligible studies will be observational studies (cohort, cross sectional, or health surveys) reporting prevalence data using validated or non-validated tools and conducted in the Ethiopian working population. Cross-sectional studies will be the most appropriate study design to determine the pooled prevalence of the work-related musculoskeletal disorders. For cohort studies, only the first phase (cross-sectional) data will be considered. We will exclude studies published in non-English, reviews, commentaries, conference abstracts, letter to editors, non-human articles, and studies conducted outside of Ethiopia.

### Study screening and selection for inclusion in the review

The titles and abstracts of articles retrieved from the search of different databases will be stored and managed in a Zotero version 5 reference manager and we will remove the duplicate records from it. Two reviewers (BJ and KN) will independently review the titles and abstract part of all the articles, the full text of the potentially eligible article will be assessed for compliance and analyzed for inclusion. Disagreements or conflicts throughout the review process will be resolved by consensus and if needed, by requesting the opinion of the third and fourth reviewers. The consistency of the selection process and quality assessment across the reviewers will be ensured by calculating the level of inter-rater agreement (Kappa statistics) [26]. A PRISMA flowchart showing details of the studies included and excluded at each stage of the study selection process will be provided.

### Data extraction and management

Once eligible studies are identified, two independent reviewers will extract the data using a prepared standardized data extraction form. Reviewers will not perform data extraction from an article of which they were (co-) authors to eliminate possible bias. Data such as first author’s last name, year of publication, study location within Ethiopia, sample size, response rate, the reason for non-response, number of events, regions of pain reported, data on prevalence, recall period, ascertainment of outcome measures, risk factor or protective factors determined by each study along with their respective odds ratio (OR) and 95% confidence interval, and information needed for the risk of bias assessment will be extracted (Table 1). The findings of the review will be illustrated through figures and tables.

**Table 1:** Data items that will be extracted

1.	Publication details: title, journal, author, year, city/region in Ethiopia, where the study was conducted, type of publication, and source of funding, if any
2.	Design: type of study design (observational studies, cross sectional, cohort, case control, others); aims or objective of the study, method of data collection, outcome variable (measures and operational definition), response rate, recruitment and sampling method, eligibility (inclusion and exclusion criteria)
3.	Study participants information’s: sample size, population characteristics including settings, role in the institution, age, gender, ethnicity, education level, demographic information’s, behavioral information’s, physical measurements, comorbidities, other health-related characteristics, employment details, work and work-environment related characteristics, bodily distribution of pain
4.	Data for outcome variables: all reported estimates, or sufficient data to calculate an estimate of the point prevalence, cumulative incidence, and incidence rate of musculoskeletal disorders in categories of bodily distribution like low back pain, neck pain, shoulder pain, elbow pain, wrist and hand pain, hip pain, knee pain, ankle pain, etc
5.	Limitations: bias (selection, response, information), limitation of outcome measures/tools, and limitation reported by authors

### Risk of bias and quality assessment

Two review authors (BJ and KN) will independently assess the quality of all included studies using the Newcastle-Ottawa Quality Assessment tool adapted for cross-sectional studies [27, 28]. The tool will be further adapted for use in this review (Additional file 3). Discrepancies of aggregate or total scores will be resolved by the third reviewer (TG) after a detailed evaluation of the source of the discrepancy. The tool contains three domains; selection of participants (3 items), quality of data (4 items), and definition of work-related musculoskeletal disorders (3 items). For the purpose of this review, all the items in the appraisal tool will be equally weighted and so the total score will be 10. There will be no subminimum score criteria for inclusion of studies.

### Data synthesis

The prevalence rate, the logarithm of prevalence, and standard error (SE) of the logarithm of prevalence will be computed. Correspondingly, for the factors associated, the logarithm of OR and SE of the logarithms of OR will be calculated. The pooled prevalence (proportion) of musculoskeletal disorders or musculoskeletal pain-related work and the pooled odds ratios (OR) of associated factors with a 95% confidence interval will be calculated using random-effects and quality effects model. The quality-effects meta-analysis [29] will be used to examine how the quality of each study influenced the pooled estimate compared with the results from the random effects [30]. The quality scores of each included study will be incorporated in the calculation of study weight to improve the robustness and help minimize the estimator variance and subjectivity in quality assessment. The presence of heterogeneity among studies will be quantified by estimating variance using both Cochrane’s Q statistics and the *I*^*2*^ statistics. *I*^*2*^statistics is the proportion of the variation in the estimates of prevalence due to genuine variation in prevalence rather than sampling error [31, 32]. Funnel plots will be used to assess small study effects or publication bias [33] by inspection of asymmetry and in addition Egger’s regression test (*p*<0.05) [34] and Beggs equations will be computed to declare publication bias. Double arsine transformation will be used in the case of variance instability [35].

Possible subgroups will be identified based on the study characteristics and population characteristics. Subgroup analysis will be performed to determine the source of heterogeneity attributed to gender, sample size, place of study, study setting, study design, year of publication, outcome tool used, type of occupation, and region of pain. Sensitivity analysis will be performed after excluding each study one by one and the pooled estimate will be calculated for the remaining studies. All statistical analyses will be performed using Meta XL version 5.3 [36] and STATA 15 Metaprop package [37]. Quantitative data from different designs, variability in prevalence (e.g., cohort, case-control studies) will not be combined, nor unadjusted and adjusted models. Transformation of rates to their natural logarithm, with corresponding standard errors (SEs) will be entered into meta-analyses. When quantitative synthesis is not feasible, we will retain the studies for narrative synthesis.

### Timeline, presenting, and reporting of the results

The review process will commence only after the final peer-review comments are received, and the protocol accepted for publication. The study selection step by step process will be outlined in a flow diagram and the reasons for exclusion will also be mentioned. The study characteristics, risk of bias, and quality assessment of the included studies will be presented in tables. Forest plots will be used to display the pooled estimates of prevalence proportions.

## Discussion

In the past decade, institutional and population-based studies on work-related musculoskeletal disorders have grown in Ethiopia. This review will aim to provide a current overview of the prevalence of musculoskeletal disorders in working-aged Ethiopian adults from various occupations, cultures, and age groups to provide a better understanding of musculoskeletal health and its effect on their life. This review will also synthesize all possible available information for occupational health policymakers, healthcare workers, and identify priority areas for interventions in work-related musculoskeletal disorders in Ethiopia.

Furthermore, this will be the first systematic review and meta-analysis that will determine the pooled prevalence of musculoskeletal disorders among the adult working population in Ethiopia. In addition, this paper will also identify factors associated with work-related musculoskeletal disorders among Ethiopians. Though WRMSD is not life-threatening, the initiative to include WRMSDs is critical in developing countries rather than being stand-alone. We believe that the inclusion of WRMSDs in Ethiopia will avoid efforts doubling and wasting of resources in the future. The rationale for the need to conduct this review is the previous two African-based reviews reporting on WRMSDs (2015) and low back pain (2007) were limited by not including any studies from Ethiopia [14, 38] and in addition, Ethiopian-specific factors like social structure, genetic diversity, nutritional status, and work culture will surely add more insight on the Ethiopian working population.

Among the potential challenges we anticipate are most importantly at review level decisions as to studies reporting the prevalence of headache should be classified as dealing with musculoskeletal disorders or not, as well as at study level by heterogeneity in occupations, measurement tools, and region of pain. We will strive to mitigate these anticipated and future challenges by highlighting decision criteria, limitations, gaps in knowledge, and caution in the interpretation of findings.

## Strengths and limitations

The strengths of this review include established national-level consensus of data, as well as a systematic and transparent approach. Our search will be conducted in close collaboration with the specialized research librarian, the screening and extraction will be performed by two researchers using a standardized extraction form. The inter-rater agreement between reviewers in the selection process will be statistically assessed. Furthermore, this review will also be the first one to consensus data on work-related musculoskeletal disorders in Ethiopia.

We anticipate that publication bias and heterogeneity may pose a limitation for this review. There are also possibilities of reporting and response set bias. In addition, this study is restricted to published reports in the English language.

## Dissemination

The findings of this review will be disseminated through publication in peer-reviewed journals and conference presentations at the relevant venue.

## Supplementary information

**Additional file 1.** PRISMA-P (Preferred Reporting Items for Systematic review and Meta-Analysis Protocols) 2015 checklist: recommended items to address in a systematic review protocol.

**Additional file 2.** Example search that will be used for screening of articles in PubMed database.

**Additional file 3.** Adapted table of Newcastle-Ottawa quality assessment tool.

## Data Availability

Not applicable

## Abbreviations

BoD: Burden of diseases
DALYs: Disability-adjusted life years
GBD: Global burden of disease
LBP: Low back pain
LMICs: Low-middle income countries
MeSH: Medical Subject Heading
MOOSE: Meta-analysis Of Observational Studies in Epidemiology
MSDs: Musculoskeletal disorders
NCDs: Non-communicable diseases
OR: Odds ratio
PRISMA-P: Preferred Reporting Items for Systematic Reviews and Meta-analysis Protocols
SDI: Socio-demographic index
SE: Standard error
WHO: World Health Organization
WRMSDs: Work-related musculoskeletal disorders
YLD: Years lived with disability
