# Supplementary material for: The prevalence and risk factors of work-related musculoskeletal disorders among adults in Ethiopia: a study protocol for extending a systematic review with meta-analysis of observational studies
Source: Syst Rev. 2020 Jun 8;9:136. doi: 10.1186/s13643-020-01403-9 (PMC7282038; doi:10.1186/s13643-020-01403-9)
Supplement: Supplementary file 2 — Additional file 2. Example search that will be used for screening of articles in PubMed database. [file 13643_2020_1403_MOESM2_ESM.docx]

**Additional file 2:**

**Example search used for identification of articles on PubMed database**

**Search #1:** ("Musculoskeletal Disorders"[Mesh]) OR "Work-related Musculoskeletal Disorders "[Mesh] OR Musculoskeletal pain OR neck pain OR spinal pain OR back pain OR low back pain OR shoulder pain OR elbow pain OR groin pain OR knee pain OR ankle pain AND Prevalence AND Ethiopia) Filters: Humans; English; Adults aged 18 & >

**Search #2:** adults AND workers OR employees OR staffs OR work place OR occupation

**Search #3:** Ethiopian* OR Ethiopia

**Search #4:** Search #1 AND Search #2 AND Search #3

Likewise, EMBASE, MEDLINE, CINAHL, Science Direct index, and Google Scholar databases will be searched using similar search terms tailored to each database
