# Supplementary material for: The prevalence and risk factors of work-related musculoskeletal disorders among adults in Ethiopia: a study protocol for extending a systematic review with meta-analysis of observational studies
Source: Syst Rev. 2020 Jun 8;9:136. doi: 10.1186/s13643-020-01403-9 (PMC7282038; doi:10.1186/s13643-020-01403-9)
Supplement: Supplementary file 3 — Additional file 3. Adapted table of Newcastle-Ottawa quality assessment tool. [file 13643_2020_1403_MOESM3_ESM.docx]

**Table 1 Critical appraisal of included studies using Newcastle Ottawa scale**

|  | **Criteria** | **Yes/No** | **Comments** |
| --- | --- | --- | --- |
| **A** | **Is the final sample representative of the target population?** |  |  |
| **1** | At least one of the following must apply in the study: an entire target population, randomly selected sample or sample stated to represent the target population. |  |  |
| **2** | At least one of the following: reasons for non-response described, non-responders described, comparison of responders and non-responders, or comparison of sample and target population. |  |  |
| **3** | Response rate, and if applicable, drop-out rate reported |  |  |
| **B** | **Quality of data** |  |  |
| **4** | Were the data primary data of musculoskeletal pain/musculoskeletal disorder, regional body pain (eg: low back pain, neck pain, upper extremity pain, lower extremity pain) or was it taken from a survey not specifically designed for that purpose? |  |  |
| **5** | Were the data collected from each subject directly or were they collected from a proxy? |  |  |
| **6** | Was the same mode of data collection used for all subjects? |  |  |
| **7** | At least 1 of the following in case of:  a) Questionnaire: a validated questionnaire or at least tested for reproducibility?  b) Interview: interview validated, tested for reproducibility, or adequately described and standardized?  c) Examination: examination validated, tested for reproducibility, adequately described and standardized? |  |  |
| **C** | **Definition of Musculoskeletal disorders/pain related to work** |  |  |
| **8** | Was there a precise anatomic delineation or condition specified or reference to an easily obtainable article that contains such specification? |  |  |
| **9** | 9. Was there further useful specification of the definition of work-related musculoskeletal pain or disorder, or question(s) put to study subjects quoted such as frequency, duration, or intensity, and character of the pain. Or was there reference to an easily obtainable article that contains such specification? |  |  |
| **10** | Did the study clearly mention the recall periods: e.g. 1 week, 1 month, life time? |  |  |
|  | **Total score (10)** |  |  |
